# Development of a neutralization monoclonal antibody with a broad neutralizing effect against SARS-CoV-2 variants

**DOI:** 10.1186/s12985-023-02230-9

**Published:** 2023-12-01

**Authors:** Hae Li Ko, Deuk-ki Lee, Younghyeon Kim, Hui Jeong Jang, Youn Woo Lee, Ho-Young Lee, Sang-Hyuk Seok, Jun Won Park, Jin-Kyung Limb, Da In On, Jun-Won Yun, Kwang-Soo Lyoo, Daesub Song, Minjoo Yeom, Hanbyeul Lee, Je Kyung Seong, Sungjin Lee

**Affiliations:** 1 Division of Research Program, Scripps Korea Antibody Institute, Chuncheon, 24341 Republic of Korea; 2 Department of Microbiology, College of Medical Science, Kangwon National University, Chuncheon-si, Gangwon-do 24341 South Korea; 3 Department of Nuclear Medicine, Seoul National University Bundang Hospital, Seongnam-si, Gyeonggi-do 13620 South Korea; 4 Division of Biomedical Convergence, College of Biomedical Science, Kangwon National University, Chuncheon-si, Gangwon-do 24341 South Korea; 5 Korea Mouse Phenotyping Center (KMPC), Seoul National University, Seoul, 08826 South Korea; 6 Laboratory of Developmental Biology and Genomics, Research Institute for Veterinary Science, and BK21 Program for Veterinary Science, College of Veterinary Medicine, Seoul National University, Seoul, 08826 South Korea; 7 Laboratory of Veterinary Toxicology, College of Veterinary Medicine, Seoul National University, Seoul, 08826 South Korea; 8 Interdisciplinary Program for Bioinformatics, Program for Cancer Biology and BIO-MAX/N-Bio Institute, Seoul National University, Seoul, 08826 South Korea; 9 College of Veterinary Medicine, Jeonbuk National University, Iksan, 54596 Republic of Korea; 10 Department of Veterinary Medicine Virology Laboratory, College of Veterinary Medicine and Research Institute for Veterinary Science, Seoul National University, Seoul, 08826 Republic of Korea

**Keywords:** Severe acute respiratory syndrome coronavirus 2, COVID-19, Antibody, Variant of concern, Human angiotensin-converting enzyme 2, Quaternary epitopes of RBD, Single-chain fragment variable

## Abstract

**Background:**

The emergence of severe acute respiratory syndrome coronavirus 2 (SARS-CoV-2) variants has challenged the effectiveness of current therapeutic regimens. Here, we aimed to develop a potent SARS-CoV-2 antibody with broad neutralizing effect by screening a scFv library with the spike protein receptor-binding domain (RBD) via phage display.

**Methods:**

SKAI-DS84 was identified through phage display, and we performed pseudovirus neutralization assays, authentic virus neutralization assays, and in vivo neutralization efficacy evaluations. Furthermore, surface plasmon resonance (SPR) analysis was conducted to assess the physical characteristics of the antibody, including binding kinetics and measure its affinity for variant RBDs.

**Results:**

The selected clones were converted to human IgG, and among them, SKAI-DS84 was selected for further analyses based on its binding affinity with the variant RBDs. Using pseudoviruses, we confirmed that SKAI-DS84 was strongly neutralizing against wild-type, B.1.617.2, B.1.1.529, and subvariants of SARS-CoV-2. We also tested the neutralizing effect of SKAI-DS84 on authentic viruses, in vivo and observed a reduction in viral replication and improved lung pathology. We performed binding and epitope mapping experiments to understand the mechanisms underlying neutralization and identified quaternary epitopes formed by the interaction between RBDs as the target of SKAI-DS84.

**Conclusions:**

We identified, produced, and tested the neutralizing effect of SKAI-DS84 antibody. Our results highlight that SKAI-DS84 could be a potential neutralizing antibody against SARS-CoV-2 and its variants.

**Supplementary Information:**

The online version contains supplementary material available at 10.1186/s12985-023-02230-9.

## Background

Severe acute respiratory syndrome coronavirus 2 (SARS-CoV-2, Wuhan Hu-1), which emerged in 2019, rapidly caused a pandemic, resulting in unprecedented global health and economic crises [1]. Since the onset of the coronavirus disease (COVID-19) pandemic, new genetic variants of SARS-CoV-2 have emerged worldwide, despite the implementation of numerous countermeasures and public quarantines. Starting with the Alpha variant (B.1.1.7), which was the first variant of concern (VOC), mutations continued to accumulate till the appearance of the current B.1.1.529 variant, known as “Omicron” [2]. As of October 2023, the B.1.1.529 sublineage EG.5.1 has become the predominant strain (https://nextstrain.org/ncov/open). B.1.1.529, classified as the fifth VOC by the World Health Organization (WHO) [3], has an increased ability to evade infection- or vaccine-generated immunity and has reduced the efficacy of antibody therapies [4]. In the mechanism underlying SARS-CoV-2 infection, the receptor-binding domain (RBD) of the spike (S) protein present on the surface glycoprotein of SARS-CoV-2 plays a key role in attachment to human angiotensin-converting enzyme 2 (hACE2). Therefore, the RBD is a potential target for the development of effective monoclonal antibodies (mAbs). However, B.1.1.529 mutants have accumulated over 30 mutations in the S protein, including 15 in the RBD. This rapid viral mutation may adversely affect the neutralizing efficacy of the mAb-based therapeutics currently being evaluated in clinical trials [5], as the mutated virus evades the therapeutic mAbs. Studies have reported the safety and efficacy of mAb-based SARS-CoV-2 therapies [6]. Since 2020, seven mAbs, namely, bamlanivimab, etesevimab, casirivimab, imdevimab, sotrovimab, cilgavimab, and tixagevimab, have been approved or received emergency use authorization from the US Food and Drug Administration (FDA) [7]. However, despite their high efficacy and good safety profiles, the development of some mAbs has been discontinued, as they do not neutralize VOCs such as B.1.1.529. In this study, we aimed to develop a human immunoglobulin G1 (IgG1) mAb that targets the RBD of the S protein of SARS-CoV-2 and its VOCs and test its neutralizing efficacy. Moreover, we performed epitope mapping and protein-denaturing binding assays to elucidate the mode of action of the identified antibody.

## Methods

### Vector construction 

The expression vector encoding hACE2 was developed by inserting the hACE2 sequence into an EF1a promoter-driven expression construct. The pMD2.G envelope plasmids encoded VSVg glycoproteins under the regulation of the CMV promoter, and psPAX2 packaging plasmids encoded gag and pol genes (Addgene, Cambridge, MA, USA). The plasmid encoding the SARS-CoV-2 S protein for pseudovirus envelope expression was a gift from Professor Paul D. Bieniasz (The Rockefeller University, New York), and plasmids encoding the SARS-CoV-2 variant S protein, such as the Delta variant (B.1.617.2, plv-spike-v8) and Omicron variant (B.1.1.529/BA.1, plv-spike-v11) envelopes, were purchased from InvivoGen (San Diego, CA, USA). The pLenti-sffv-NanoLuc-PGK-RFP-T2A-PURO Lentiviral Reporter Plasmid was purchased from ALSTEM (Richmond, CA, USA). All reagent information was described in detail in the supplementary additional materials.

### Cell culture

We cultured 293T, Huh7, VeroE6, and Caco-2 cells (KCLB, Seoul, Korea) in monolayers as described previously [8] in DMEM supplemented with 1% L-glutamine, 1% penicillin–streptomycin, 1% non-essential-amino acid (Cytiva, Marlborough, MA, USA), and 10% fetal bovine serum (FBS; Thermo Fisher Scientific, Gibco, Waltham, MA, USA). Raji (ATCC, CCL-86) and U937 cells (KCLB, 21593.1) were cultured in RPMI-1640 medium supplemented with 1% L-glutamine, 1% penicillin–streptomycin, 1% non-essential-amino acid (Cytiva), and 10% FBS (Thermo Fisher Scientific). ExpiCHO-S cells (Thermo Fisher Scientific) were maintained in the ExpiCHO™ Expression Medium. All cells were maintained in a humidified shaking incubator at 37 °C in a 5% CO_2_ atmosphere and sub-cultured twice a week at a density of 0.2 × 10^6^ cells/mL.

### Phage display for antibody screening

OPAL, the human synthetic single-chain fragment variable (scFv) library expressed with scFv and HA tags with 7.6 × 10^9^ diversity, was provided by Ewha Womans University [9]. The antibody library was transformed into ER2738 *Escherichia coli* cells and incubated in 2 × YT medium containing 50 μg/mL carbenicillin and 2% glucose at 37 °C for 2 h. After 2 h, helper phages (multiplicity of infection (MOI) = 20) were treated and infected at 37 °C for 1 h, followed by centrifugation at 2900 × *g*, 30 °C for 20 min. The collected pellets were transferred to 2 × YT medium containing 50 µg/mL carbenicillin and 70 µg/mL kanamycin and incubated overnight at 30 °C. The phages were extracted using a polyethylene glycerol (PEG; Sigma-Aldrich, St. Louis, MO, USA)-based precipitation method. The phages displaying scFv were harvested for bio-panning to screen the SARS-CoV-2 Delta (B.1.617.2) variant RBD-binding scFv. Briefly, B.1.617.2 RBD was conjugated with epoxy magnetic beads (Invitrogen, Waltham, MA, USA), incubated with the phage library, and washed thrice with 0.1% PBS-T. Next, B.1.617.2 RBD-bound phages were eluted and transformed into freshly cultured ER2738 *Escherichia coli* cells. Bio-panning was performed by repeating five rounds, antibody candidates were selected through enzyme-linked immunosorbent assay (ELISA), and the complementarity-determining region (CDR) was confirmed by sequencing (Macrogen, Seoul, Korea).

### Production of humanized IgG antibodies and of neutralizing antibodies

The scFv coding sequence with confirmed scFv-SARS-CoV-2 RBD (wild-type [WT], B.1.617.2, and B.1.1.529)-binding ability was amplified using forward and reverse primers containing restriction sites for insertion into a two-vector human IgG system (heavy and light chain human IgG vectors).

Briefly, each VH primer (FW: 5′-TCGCGCGCACTCCGAGGTGCAGCTGTTG-3′; RV: 5′-CACGTCTCTATGCTGAGCTCACGGTGAC-3′) was designed with BspE1 and Bsa1 restriction sites at the 5′- and 3′-ends, respectively, and VL primers (FW: 5′-AGCCTCTCCGGACAGTCTGTGCTGACT-3′, RV: 5′-CAGGTCTCGTTCGTAGGACCGTCAGCT-3′) possessed the BssH11 and BsmB1 restriction sites at the 5′- and 3′-ends, respectively. The amplified VH fragment was inserted into the cloning site of the TGEX-HC vector, and the VL domain was ligated into the cloning site of the TGEX-LC vector (Antibody Design Labs, San Diego, CA, USA). *Escherichia coli* DH5α-competent cells (RH618; RBC Bioscience, New Taipei, City, Taiwan) were used for plasmid amplification, and all clones were confirmed via restriction mapping and DNA sequencing (Macrogen). Heavy and light chain IgG plasmids were co-transfected into ExpiCHO-S cells according to the manufacturer’s instructions (A29127; Thermo Fisher Scientific, Waltham, MA, USA). We purified human IgG using protein A agarose resin (Amicogen, Jinju, Korea) and an affinity chromatography column (Bio-Rad Laboratories, Hercules, CA, USA).

### ELISA analysis

ELISA analysis was performed as described previously [10]. The 96-well half area plates (Corning Life Sciences, Corning, NY, USA) were coated with WT, B1.617.2 and B.1.1.529 spike proteins (R&D systems, MN, USA) containing 1 × PBS (30 ng/ 30 μL per well) overnight at 4 °C, respectively. After washing three times with 0.05% PBS-T, the coated wells were blocked and incubated with anti-SARS-CoV-2 antibodies for 1 h at room temperature. After washing, 30 μL of 0.8 mg/mL HRP-conjugated anti-human antibody was added and the plate was incubated for 1 h room temperature. After three times washing, tetramethylbenzidine substrate was added at room temperature, and the reaction was stopped with 2N H_2_SO_4_. Absorbance was read at 450 nm on microplate reader (Promega, Madison, WI, USA).

### Generation of stable hACE2 cell lines

To establish Huh7-hACE2-Puro cells, cells containing lenti-*hACE2* transfer plasmids and psPAX2 packaging plasmids were co-transfected into HEK293T cells with the corresponding envelope plasmid (pMD2.G). HEK293T cells at 80% confluence in a T-75 flask were transfected with Opti-MEM with lipoplexes containing transfer plasmids (8 μg), envelope plasmids (8 μg), psPAX2 plasmids (8 μg), Plus Reagent (100 μL; Life Technologies, Carlsbad, CA, USA), and Lipofectamine 3000 (50 μL). After 24 h, the medium was replaced with DMEM supplemented with 10% FBS. Briefly, the virus-containing medium was harvested 72 h after transfection, centrifuged at 1500 × *g* for 10 min, and subsequently pre-cleaned using 0.45-μm filters (Merck Millipore, Burlington, MA, USA). Huh7 cells (1 × 10^4^/plate) were transduced with 1 MOI hACE2/Puro vectors. After 24 h, the medium was replaced with fresh medium, and the plates were incubated for 72 h. The infected cells were supplemented with puromycin (final concentration: 10 μg/mL). The selection medium was replaced every 3 days for 2 weeks.

### Packaging of SARS-CoV-2 variant pseudoviruses

SARS-CoV-2 wild-type (WT) and B.1.617.2 or B.1.1.529 variant pseudoviruses were produced as previously described [11]. To produce SARS-CoV-2 wild-type (WT) and B.1.617.2 or B.1.1.529 variant pseudoviruses, HEK293T cells were transfected when they reached 80% confluence in a T-75 flask. The transfection mixture consisted of pLenti-sffv-NanoLuc-PGK-RFP-T2A-PURO Lentiviral Reporter Plasmid (14 μg), envelope plasmids encoding SARS-CoV-2 S protein (8 μg), psPAX2 plasmids (8 μg), and Lipofectamine 3000 (75 μL) in Opti-MEM. After 24 h, the medium was replaced with DMEM supplemented with 10% FBS. The pseudovirus-containing medium was harvested 72 h after transfection, centrifuged at 500 × g for 10 min, and subsequently pre-cleaned using 0.45-μm filters (Merck Millipore, Burlington, MA, USA). The clarified supernatant was then mixed with 1/3 volume of Lenti-X Concentrator (Takara Bio, Japan) and incubated at 4 °C for 1 h. After incubation, the mixture was centrifuged at 1500 × g for 45 min at 4 °C, and an off-white pellet was resuspended with 1 mL of PBS. The resuspended pseudovirus was stored at − 70 °C until used.

### SARS-CoV-2 variant pseudovirus neutralization assays

Huh7-hACE2 cells were infected with a lentivirus-based nano-luciferase expressing SARS-CoV-2 pseudovirus for neutralization antibody candidate screening. Luciferase activity was measured as a surrogate for pseudovirus neutralization levels. Thirteen neutralizing antibodies with cross-reactivity were first tested at a single concentration of 1 μg/mL for 72 h for binding to SARS-CoV-2 variants, namely WT, B1.617.2, and B.1.1.529. Furthermore, 1 × 10^4^ cells (hACE2-Huh7) were plated on a white 96-well plate (Costar 3610; Corning Life Sciences, Corning, NY, USA), co-incubated with pseudovirus (1 MOI) and the serially diluted antibody (PBS or 1, 10, 100, 1, 10, or 100 μg/mL) for 1 h at 37 °C, and added to the wells. After 24 h, the medium was replaced with fresh medium, and the plate was incubated for 72 h. After 72 h, the cells were incubated for 3 h at 37 °C in the presence of EZ-CYTOX reagent (10% tetrazolium salt; Dogenbio, Seoul, Korea) for cytotoxicity assessment. Nano-luciferase activity was measured using a nano-luciferase reagent (Promega, Madison, WI, USA).

### Surrogate neutralization assays

SARS-CoV-2 surrogate virus neutralization test kit was obtained from Genscript (NJ, USA) and the tests were carried out according to the manufacturer’s instructions. The Antibody (SKAI-DS10, 64, 84 and Bamlanivimab) was diluted 1:10 and mixed with an equal volume of HRP-conjugated to B.1.617.2 RBD or B.1.1.529 RBD (6 ng) and incubated for 30 min at 37 °C. A 100 μl volume of each mixture was added to each well on the microplate coated with ACE-2 receptor. The plate was sealed and incubated at room temperature for 15 min at 37 °C. After washing three times with wash solution, 100 μl tetramethylbenzidine substrate was added each well and incubated in the dark at room temperature for 15 min. The reaction was stopped by addition of 50 μl stop solution to each well and the absorbance was read at 450 nm on microplate reader (Promega, Madison, EI, USA).

### Quantitative reverse transcription PCR (RT-qPCR) analysis

Vero E6 cells (1 × 10^5^) were plated in a 6-well plate (Costar 3610; Corning Life Sciences, Corning, NY, USA). B.1.617.2 or B.1.1.529 was incubated with the SKAI-DS84 antibody at the indicated concentrations (tenfold serial dilution) for 1 h at 37 °C and then added to the wells of the 6-well plate seeded with Vero E6 cells.

Three days after incubation, total cellular RNA was extracted using an RNeasy® mini kit (Qiagen, Hilden, Germany) per the manufacturer’s instructions. The yield of the extracted RNA was assessed spectrophotometrically. The expression of B.1.617.2 or B.1.1.529 RNA and cellular RNAs was measured using RT-qPCR. The expression of each gene was normalized to that of the endogenous reference gene glyceraldehyde-3-phosphate dehydrogenase (GAPDH). DNA quantification was performed using a QuantStudio3 real-time PCR detection system (Applied Biosystems, Waltham, MA, USA). The primers used for RT-qPCR were as follows: FW-SARS-CoV-2-B.1.617.2: CCACAAAAACAACAAAAGTTGG, RV-SARS-CoV-2-B.1.617.2: TGAGAGACATATTCAAAAGTGCAA, FW-SARS-CoV-2-B.1.1.529: GGACCCTCAGATTCAACTGG, RV-SARS-CoV-2-B.1.1.529: GCAGTATTATTGGGTAAACCTTGG, FW-GAPDH: TGGTCTCCTCTGACTTCA, and RV-GAPDH: CGTTGTCATACCAGGAAATG.

Tissues were weighed and homogenized with zirconia beads in a MagNA Lyser instrument (Bio-Rad Laboratories) in 10 mL of DMEM supplemented with 2% heat-inactivated FBS. Tissue homogenates were clarified by centrifugation at 10,000 × *g* for 5 min and stored at − 80 °C. RNA was extracted using a MagMax mirVana total RNA isolation kit and a Kingfisher duo prime extraction machine (Thermo Fisher Scientific). The SYBR Green RT-qPCR assay (one step) was performed according to the manufacturer’s instructions using a QuantiFast SYBR® Green RT-PCR kit (Qiagen); 8 μL of target RNA and 1 μL [10 pM/μL] of each primer (L Primer: CCCTGTGGGTTTTACACTTAA; R primer: ACGATTGTGCATCAGCTGA; probe: 5'-FAM-CCGTCTGCGGTATGTGGAAAGGTTATGG-BHQ1-3) were added to 12.5 μL 2 × Master Mix QuantiFast SYBR Green RT-PCR, 0.25 μL of enzyme RT-Mix, and Ultra-Pure Water (Thermo Fisher Scientific) (final reaction volume: 25 μL). The reverse transcription reaction conditions were as follows: 50 °C for 10 min, 95 °C for 5 min, and 40 cycles of 95 °C for 10 s and 60 °C for 30 s. The reaction was completed by determining the dissociation curve of all amplicons generated using an ABI 7500 device (Applied Biosystems).

### Animal experiments

Animal experiments were performed as described previously [12]. For animal experiments, 8-week-old male B6.Cg-Tg(K18-ACE2)2Prlmn/J mice (The Jackson Laboratory, CA, USA) were housed in a certified animal biosafety level 3 (ABSL3) facility at the Ji Seok-Yeong Biomedical Research Institute of Seoul National University Bundang Hospital in Seongnam, Republic of Korea. All procedures were approved by the Institutional Animal Care and Use Committee (Approval No. BA-2108-325-078), and the Institutional Biosafety Committee of Seoul National University Bundang Hospital approved biosafety experimental protocols (Approval No. IBC-2105-A-008). The hACE2-transgenic (TG) mice (five mice in each group) were intranasally inoculated with 50 μL of Omicron variant virus (1 × 10^5^ PFU) under anesthesia. After four hours of infection, either PBS or 50 mg/kg of SKAI-D84 was intravenously injected. Lung, spleen and duodenum tissues were harvested from hACE2-TG mice two and seven days after SARS-CoV-2 Omicron variant infection, and viral RNA was quantified using RT-qPCR.

The B.1.1.529 strain (accession number: NCCP43408) was purchased from the Korea Centers for Disease Control and Prevention (KDCDC03/2020) and Vero E6 cells (CRL-1586) from the Korea Microbial Resource Center (KCTC). All experiments with SARS-CoV-2 were performed at the Biosafety Level 3 (BSL3) Laboratory of the Seoul National University Bundang Hospital.

### Virus quantification (TCID_50_)

The tissue culture infectious dosage (TCID_50_) was determined utilizing the Reed–Muench technique. Vero E6 cells were allocated in 12-well plates at a density of 3 × 10^5^ cells per well, and allowed to form a monolayer by the day before the plaque assay. The cells underwent a one-hour infection in duplicate with tenfold serial dilutions of B.1.512, and were then covered with a 0.3% SeaPlaque (LONZA, Basel, Switzerland) agarose medium inclusive of 2% FBS. Post a 72 h incubation period, the virus-infected cells were fixed with a 4% paraformaldehyde solution for an hour, followed by staining using a crystal violet solution (548-62-9, Sigma–Aldrich, St. Louis, USA). The viral titers were quantified in plaque-forming units (PFU) per milliliter.

### Histopathology analysis

Lung and spleen tissues were fixed in 10% neutral buffered formalin for 1 day. Paraffin-embedded sections (3 µm) were stained with hematoxylin and eosin (H&E). The lesions were graded using a semi-quantitative scale based on the percentage of tissue affected by pathological changes as follows: 0: absent; 1: minimal, less than 10% of tissue affected; 2: mild, more than 10% but less than 25% of tissue affected; 3: moderate, more than 25% but less than 50% of tissue affected; 4: moderately severe, more than 50% but less than 75% of tissue affected; and 5: severe, more than 75% of tissue affected. Pulmonary lesions were evaluated based on the presence and abundance of the following symptoms: (1) pulmonary inflammation, involving the perivascular/peribronchial spaces with a moderate number of inflammatory cells surrounding the regions and interstitial spaces with more than five inflammatory cells in each alveolar space; (2) pulmonary edema, involving the perivascular spaces exhibiting edematous cuffs and alveolar edematous spaces. Splenic lesions were evaluated based on the scoring for apoptosis in the white pulp, which was determined based on the extent of the area affected. The scoring of white pulp atrophy was based on the extent of size reduction compared with that of the normal splenic white pulp. The histopathological scores were determined by two veterinary pathologists.

### Antibody dependent enhancement assay

For antibody-dependent enhancement analysis, lentivirus-based SARS-CoV-2 pseudovirus expressing nano-luciferase was used to infect Huh7-hACE2, Raji, and U937 cells. Luciferase activity was measured as a surrogate for pseudovirus infection levels. SKAI-DS84 was tenfold serially diluted from 100 to 2 × 10^–5^ μg/ml and pre-incubated for 1 h with pseudovirus SARS-CoV-2 WT at 1 MOI. In parallel, 1 × 10^4^ cells (hACE2-Huh7, Raji, and U937) were plated in white 96-well plates (Costar 3610; Corning Life Sciences, Corning, NY, USA), and the virus pre-mixed with SKAI-DS84 was added to the wells. After 24 h, the medium was replaced with fresh medium, and the plates were incubated for 72 h. Following 72 h, nano-luciferase activity was measured using the Nano-Glo Luciferase Assay System (Promega, Madison, WI, USA).

### Surface plasmon response (SPR) assay

B.1.1.529.1 (BA1) or B.1.1.529.2 (BA2) proteins (3 µg/mL) in 10 mM sodium acetate (pH 4.5) were covalently immobilized on a CM5 chip (Cytiva, Marlborough, MA, USA). Immobilization of the captured protein was achieved by standard amine coupling chemistry (Cytiva); the flow cells were activated with a 1:1 mixture of 0.4 M 1-ethyl-3-(3-dimethylaminopropyl)-carbodiimide (EDC) and 0.1 M N-hydroxysuccinimide (NHS) at a flow rate of 30 µL/min. Reference flow cells were used as blanks and activated as previously described [13]. BA1 and BA2 proteins were mixed with sodium acetate (pH 4.5) and target (100 RU), resulting in a surface density of approximately 240 RU. Both surfaces were blocked by injecting 1 M ethanolamine/HCl (pH 8.5). The SKAI-DS84 antibody was injected into both flow cells at 25, 12.5, 625, 3.13, 1.56, or 0.78 nM at a flow rate of 30 µL/min, contact time of 120 s, and dissociation time of 600 s. A regeneration solution comprising 10 mM glycine (pH 2.1) was injected for 30 s at a flow rate of 30 µL/min. The values obtained for reference flow cells were subtracted from the analyte binding responses, and the final data for both flow cells were analyzed using BIA evaluation 2.0 software (Biacore; Cytiva) in a 1:1 binding of globally fitting data to derive kinetic and equilibrium parameters.

### Protein thermal shift (PTS) analysis

PTS analysis was performed according to the manufacturer’s instructions (Invitrogen, Waltham, MA, USA), following the binding of three SKAI-DS (10, 63, and 84) antibodies to each of the B.1.617.2 and B.1.1.529 variant RBD antigens. All individual experimental data were analyzed using the recommended Protein Thermal Shift™ Software v1.4 (Thermo Fisher Scientific).

## Antibody-antigen docking modeling.

Docking was performed using Hex 8.0 ClusPro 2.0 server (https://cluspro.bu.edu/publications.php). This scrutiny was used to determine the interaction and orientation between the two molecules to determine the correct binding between the antigen and the antibodies. These software was selected from those considered in the CAPRI project (http://www.ebi.ac.uk/msd-srv/capri). The prediction of the interactions of structures was to use the main software setup, with the explanation that was used in ClusPro software using antibody prediction method.

### Statistical analysis

Two-way analysis of variance was used to assess significant differences among treatment groups. All values were expressed as mean ± standard deviations (SD); representative experiments were performed in triplicate or quadruplicate. If significant deviations from variance homogeneity, then non-parametric Bonferroni posttests was conducted. For in vivo results, unpaired Student’s t-test was used to determine significance between two groups. Statistical analyses were conducted using Prism v5.0c software (GraphPad Software, La Jolla, CA, USA). Differences were considered significant at *p* < 0.05.

## Results

### Antibodies targeting the RBD of the SARS-CoV-2 VOC were identified

To identify the antibody clones that bind to the RBD of the SARS-CoV-2 S, the OPAL scFv antibody library [9] was used for panning against B.1.617.2 RBD antigens. Bio-panning was performed for five rounds, and scFv clones were enriched against B.1.617.2 RBD antigens (Additional file 1: Fig. S1a). We confirmed that scFv could bind to WT and B.1.617.2 S protein RBD in the 4th and 5th round of panning and that it demonstrated antibody diversity (Additional file 1: Fig. S1b). Moreover, 400 individual clones that were used to express RBD-targeting scFv using isopropyl β-D-1-thiogalactopyranoside were recovered. Furthermore, for antibody selection, only individual colonies with binding ratio ≥ tenfold for WT-RBD/BSA (blue), B.1.617.2- RBD/BSA (red), or B.1.1.529/BSA (green) were selected (Additional file 1: Fig. 1c). Of the selected scFv clones, 13 neutralizing antibody candidates were selected after sequence analysis, and heavy and light chain Complementarity-Determining Region 1(CDR), CDR2, and CDR3 antibody sequences were identified. These SARS-CoV-2 VOC RBD-binding scFv sequences were cloned into a human IgG1 vector system to evaluate their neutralizing effects.

**Fig. 1:**
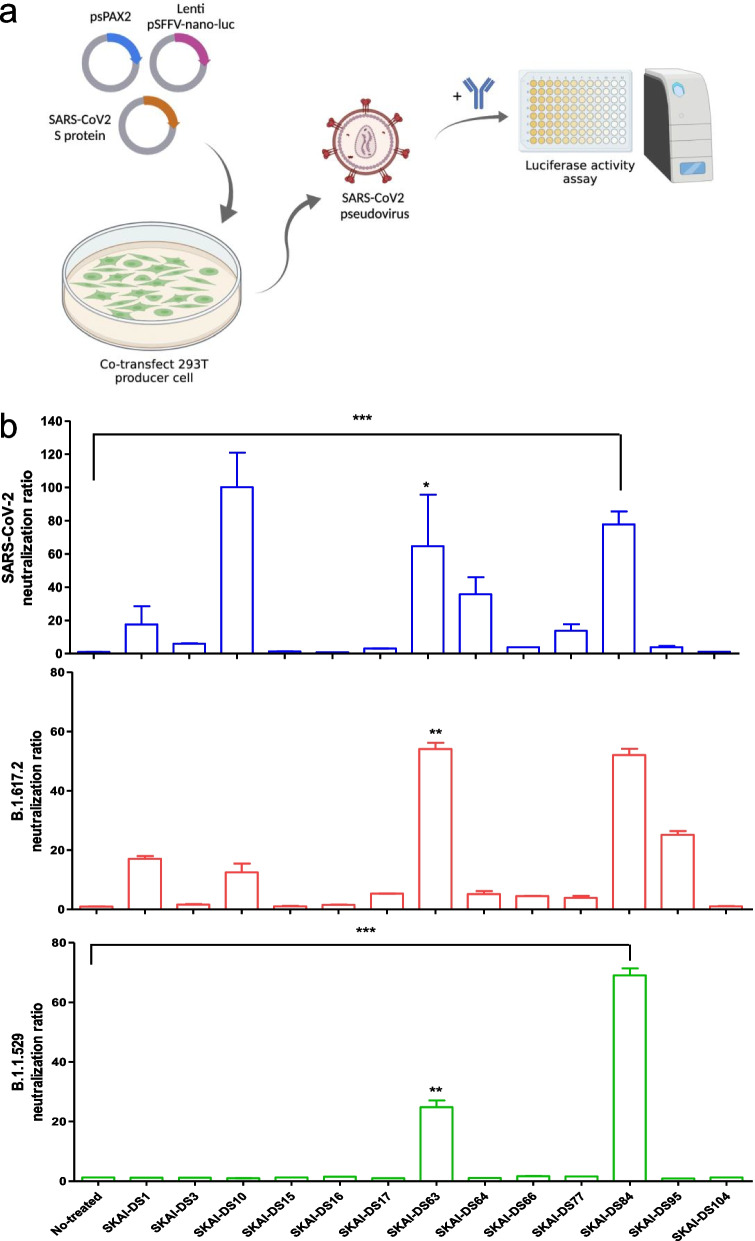
Screening for neutralization antibodies against SARS-CoV-2 and variants. **a** Thirteen IgG1 antibodies were synthesized from the OPAL antibody library, and their neutralization effects on SARS-CoV-2 were evaluated using Nluc-SARS-CoV-2 pseudoviruses (SARS-CoV-2_pp_). **b** Thirteen (10 µg/mL) antibodies were pre-incubated with WT or variant SARS-CoV-2_pp_ (B.1.617.2 or B.1.1.529/BA.1) for 1 h. After incubation for 1 h, ACE2-expressing cells were infected with SARS-CoV-2 WT and variants. Their effects on neutralization were assessed via luciferase analysis, and cell viability was measured using a WST-based cytotoxicity assay. WT, wild-type; SARS-CoV-2, severe acute respiratory syndrome coronavirus 2. *P < 0.05, **P < 0.01, significantly different from control

### SKAI-DS84 is a potent cross-neutralizing mAb against SARS-CoV-2 pseudoviruses

To verify the neutralizing ability of the selected neutralizing antibody candidates, WT, B.1.617.2, and B.1.1.529 SARS-CoV-2 pseudovirus systems were constructed (Fig. 1a) [11]. For antiviral activity and cell viability screening, the 13 antibodies were individually incubated with pseudoviruses (WT, B.1.617.2, and B.1.1.529) simultaneously. After 1 h, the incubated viruses with 13 antibodies were incubated with hACE2-overexpressing Huh7 cells. Nano-luciferase activity was used as a surrogate measure for SARS-CoV-2 infection levels, and a cell proliferation assay was performed to assess cell viability. We evaluated the neutralizing activity of the 13 neutralizing antibody candidates at a concentration of 1 μg/mL against the WT, B.1.617.2, and B.1.1.529 variants. In the neutralization assays against WT, and B.1.617.2, SKAI-DS10 showed the highest neutralizing activity, followed by SKAI-DS63 and SKAI-DS84. However, in the neutralization evaluation against B.1.1.529, SKAI-DS84 exhibited the most potent neutralizing activity, while SKAI-DS63 showed moderate neutralizing activity. Surprisingly, the SKAI-DS10 antibody, which demonstrated the strongest neutralizing activity against WT and B.1.617.2, showed no neutralizing efficacy against B.1.1.529 (Fig. 1b). However, our emphasis was placed on these three antibodies for subsequent analyses. Surrogate neutralization assays were performed to evaluate their dose-dependent neutralizing activity against the B.1.617.2 and B.1.1.529 variants. Ultimately, we found that SKAI-DS84 exhibited the most potent neutralizing activity with the lowest EC_50_ against B.1.617.2 (Additional file 1: Fig. S2a) and B.1.1.529 variants (Additional file 1: Fig. S2b).

**Fig. 2:**
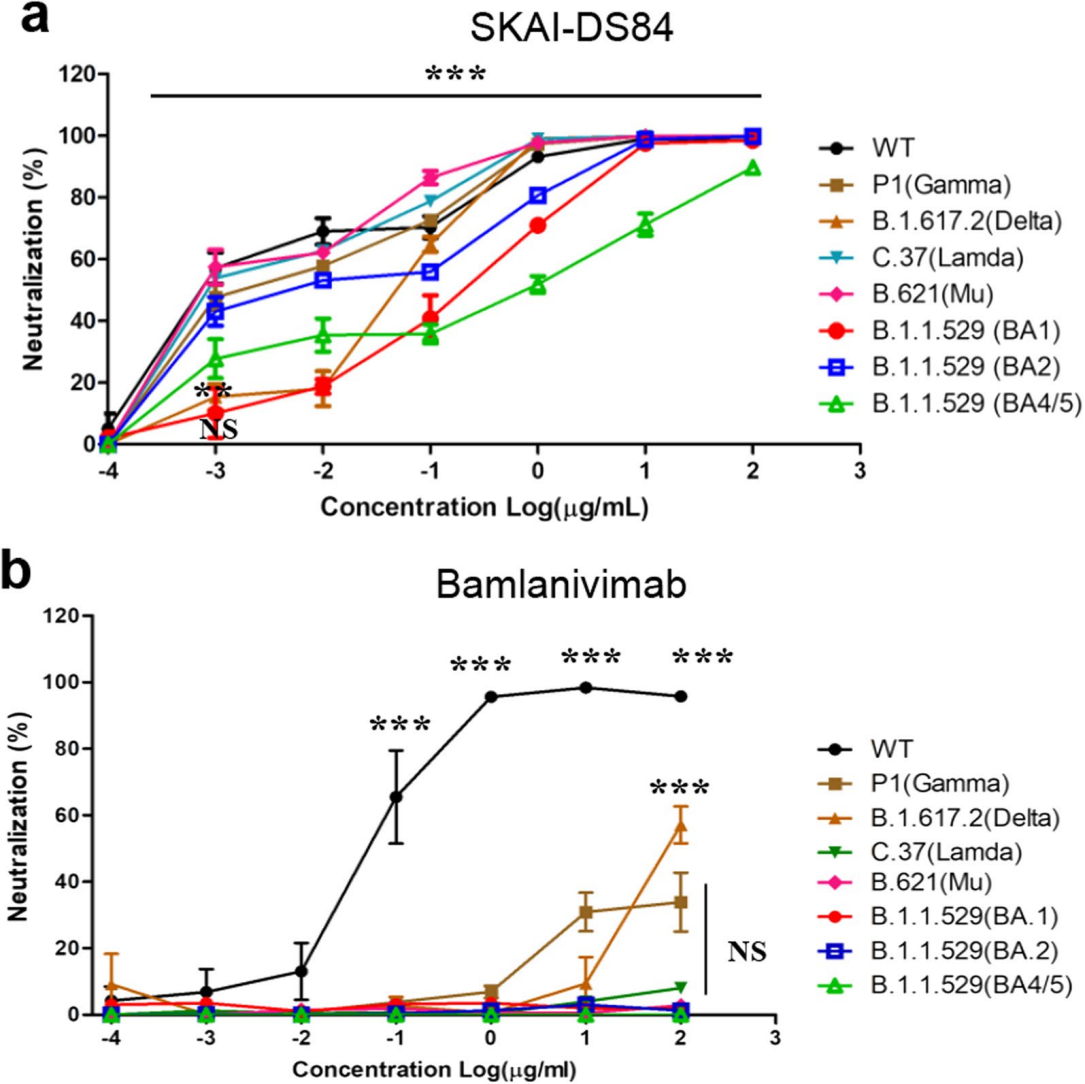
Effect of SKAI-DS84 on SARS-CoV-2 pseudo-particle neutralization. Dose–response curves were generated by measuring the relative luciferase activities of SARS-CoV-2 WT and seven variants of pseudovirus-infected hACE-Huh7 cells treated with increasing concentrations of **a** SKAI-DS84 and **b** bamlanivimab for 72 h. WT, wild-type; SARS-CoV-2, severe acute respiratory syndrome coronavirus 2. *P < 0.05, **P < 0.01, significantly different from control

Since the validation outcomes from the surrogate neutralization assays showed that SKAI-DS84 had potent neutralizing efficacy against not only the WT but also other variants, our attention was directed towards SKAI-DS84 for subsequent investigations. We evaluated the neutralizing efficacy of SKAI-DS84 in a dose-dependent manner against not only the WT but also VOC pseudoviruses (Fig. 2a). When compared with LY-CoV555 (bamlanivimab), SKAI-DS84 exhibited potent neutralizing efficacy against all the VOCs we tested. The WT demonstrated an EC_50_ of 0.002 μg/mL, while the B.1.617.2 and B.1.1.529 BA.1 variants showed EC_50_ values of 0.045 and 0.164 μg/mL, respectively. Furthermore, when we performed neutralization assays against the current VOCs—B.1.1.529 BA.2 and BA.4/5 variants—we observed EC_50_ values of 0.0124 and 0.3217 μg/mL, respectively. Compared with that against the BA.1 variant, neutralizing efficacy against BA.2 was stronger, whereas that against BA.4/5 was lower (Table 1).

**Table 1:** EC50 values for SKAI-DS84 and bamlanivimab based on SARS-CoV-2 WT and variant luciferase-based pseudovirus assays

Variants		
Antibody	SKAI-DS84	Bamlanivimab
	EC_50_ (μg/mL)	EC_50_ (μg/mL)
WT	0.002	0.005
P1 (Gamma)	0.004	> 10
B.1.617.2 (Delta)	0.0465	> 10
C.37 (Lambda)	0.0026	> 10
B.621 (Mu)	0.0025	> 10
B.1.1.529 (BA1)	0.1643	> 10
B.1.1.529 (BA2)	0.0124	> 10
B.1.1.529 (BA4/5)	0.3217	> 10

SKAI-DS84 possessed the highest inhibitory activity against WT and variant pseudoviruses. Because of its significant broad-spectrum antiviral efficacy against not only WT and B.1.617.2 but also B.1.1.529, we directed our attention to SKAI-DS84 in subsequent experiments.

### SKAI-DS84 is responsible for inhibiting WT and SARS-CoV-2 variant infection

**Fig. 3:**
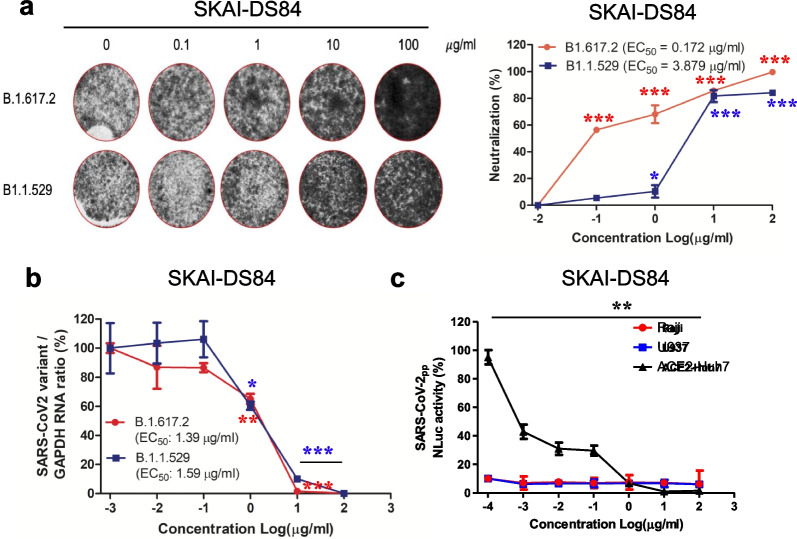
Effect of SKAI-DS84 on authentic virus neutralization. Dose-dependent effects of SKAI-DS84 on SARS-CoV-2 variant (B.1.617.2 and B.1.1.529) neutralization. **a** Crystal violet staining data obtained by measuring the TCID_50_ in authentic virus-infected VeroE6 cells treated with increasing concentrations of SKAI-DS84 for 72 h. **b** Dose–response graphs generated by measuring the relative SARS-CoV-2 and GAPDH RNA levels using RT-qPCR analyses of either B.1.617.2- or B.1.1.529-infected VeroE6 cells treated with increasing concentrations of SKAI-DS84 for 72 h. **c** The in vitro ADE assay was performed as described in Raji (FcγR II-dependent) and U973 (FcγR I&II-dependent) cells. SARS-CoV-2, severe acute respiratory syndrome coronavirus 2; ADE, antibody-dependent enhancement; TCID_50_, tissue culture infective dose. *P < 0.05, **P < 0.01, significantly different from control

To rule out the possibility of an artificial effect in the pseudovirus-based assay, we retested the antiviral effect of SKAI-DS84 on authentic viruses (WT, B.1.617.2, and B.1.1.529) [14]. We first conducted a serum neutralization assay on SARS-CoV-2 WT using the three initially discovered antibodies (SKAI-DS10, SKAI-DS63, and SKAI-DS84). The results showed that SKAI-DS10 exhibited the highest neutralizing activity with an SN factor of 3840, followed by SKAI-DS84 (SN = 120) and SKAI-DS63 (SN = 20) (Additional file 1: Fig. S2c). Based on the previous pseudovirus analysis and neutralization efficacy evaluation against the SARS-CoV-2 WT, we selected SKAI-DS84 as a potential neutralizing antibody candidate for B.1.617.2 and B.1.1.529 variants. We validated its neutralizing capability using CPE (cytopathic effect) assessment, which allows us to assess the antibody's neutralization efficacy by measuring cell survival rates based on the extent of infection with actual B.1.617.2 and B.1.1.529 viruses. SKAI-DS84 exhibited an EC50 of 0.172 μg/mL in the B.1.617.2 variant and 3.879 μg/mL in the B.1.1.529 variant. Consequently, we confirmed its ability to neutralize the virus and decrease CPE in a concentration-dependent manner. (Fig. 3a). Furthermore, quantitative reverse transcription-polymerase chain reaction (RT-qPCR) analysis demonstrated dose-dependent inhibition of B.1.617.2 (EC_50_ = 1.39 μg/mL) and B.1.1.529 (EC_50_ = 1.59 μg/mL) replication by SKAI-DS84 (Fig. 3b).

Collectively, these data suggested that SKAI-DS84 is a neutralizing antibody responsible for inhibiting infection by WT and SARS-CoV-2 variants. To determine whether SKAI-DS84 exerted antibody-dependent enhancement (ADE) effects [15], we performed an ADE assay using WT pseudovirus and Raji and U937 cells. FcγRII-expressing Raji cells and FcγRI- and FcγRII-expressing U937 cells were infected with SARS-CoV-2 pseudovirus treated with SKAI- DS84 [16]. The SKAI-DS84 antibody did not exhibit any ADE of viral infections in Raji and U937 cells (Fig. 3c). This data suggests that for SKAI-DS84, there is no ADE effect, indicating that there are no adverse effects of the antibody worsening the infection.

### SKAI-DS84 neutralized the B.1.1.529 variant in vivo

**Fig. 4:**
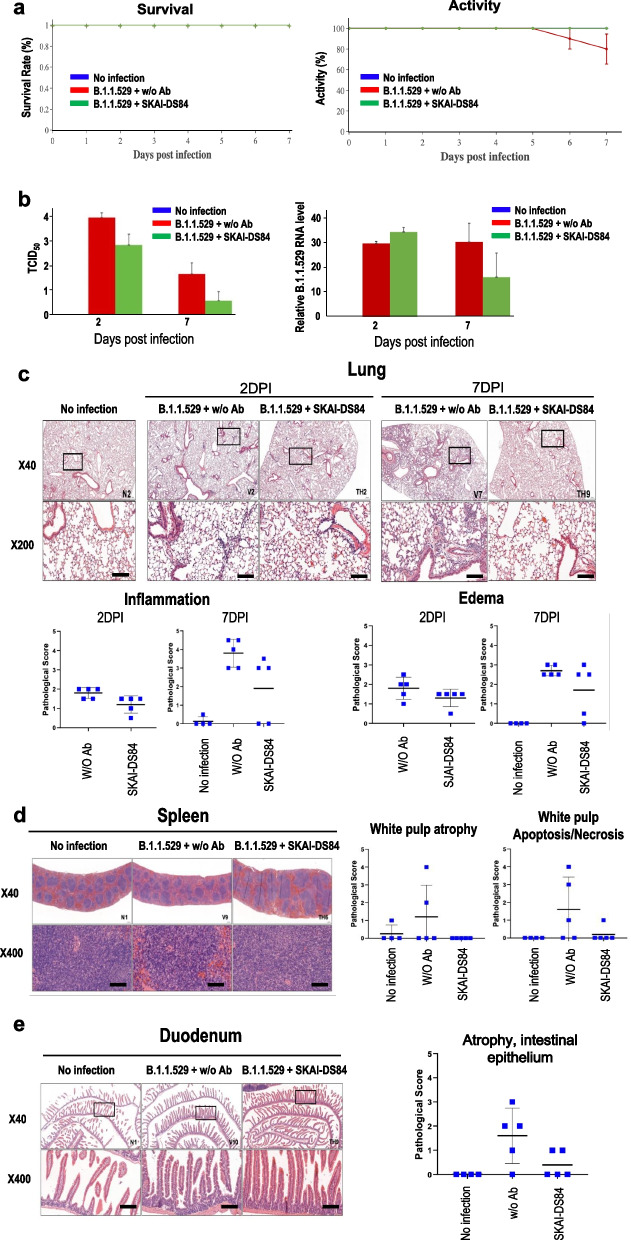
Therapeutic efficacy of SKAI-DS84 in B.1.1.529-infected K18-hACE2 expressing mice. After infecting K18-hACE2 mice with 10^5^ PFU of B.1.1.529 virus, 50 mg/kg SKAI-DS84 was intravenously injected within 4 h. All experiments were analyzed 2 and 7 days after viral infection. **a** Monitoring the survival and activity of infected mice. **b** Measurement of viral copies using RT-qPCR and TCID_50_. Histopathological analysis of SKAI-DS84-treated or untreated mice infected with B.1.1.529: **c** lung, **d** spleen, **e** duodenum. *P < 0.05, **P < 0.01, significantly different from control. Scale bar = 100 μm

To verify the neutralizing effect of SKAI-DS84 on B.1.1.529 in vivo, three groups of 4-week-old K18-hACE2 transgenic male mice (eight mice in each group) were intranasally inoculated with B.1.1.529 (1 × 10^5^ plaque-forming units (PFU)]. After 4 h, SKAI-DS84 was intravenously injected into the B.1.1.529-infected mice once, and the pathogenicity and virus titer were monitored on 2 and 7 days post-infection (dpi), respectively. A previous study reported that SARS-CoV-2 WT infection causes severe interstitial pneumonia and animal death on 7 dpi [17]. However, the survival rate of non-infected, B.1.1.529-infected, and B.1.1.529-infected mice treated with SKAI-DS84 did not decrease. The body weight and activity of the mice after SKAI-DS84 treatment were comparable as those of the PBS group, The B.1.1.529-infected mice exhibited a significant decrease in mice activity such as movement; however, the activity of the SKAI-DS84 injected group was shown to a level similar to that of non-infected mice, thereby demonstrating that SKAI-DS84's neutralization capability progressively restored mouse activity to a stable condition. To confirm the antiviral activity of SKAI-DS84 in B.1.1.529-infected mice, we performed RT-qPCR on lung and determined the tissue culture infective dose (TCID_50_). RT-qPCR analyses confirmed that B.1.1.529 replication was abrogated by SKAI-DS84 on 7 dpi. However, SKAI-DS84 did not decrease viral RNA levels at 2 dpi. Administration of SKAI-DS84 at its TCID_50_ (50 mg/kg) led to significantly reduced rates of B.1.1.529 infection on 2 and 7 dpi. Collectively, these data suggest that SKAI-DS84 plays a major role in inhibiting SARS-CoV-2 re-entry via viral neutralization. We confirmed the ability of SKAI-DS84 to ameliorate lesions in the lungs, spleen, and duodenum based on the reductions in viral RNA levels. For histological analysis, five animals from each group were euthanized 2 and 7 dpi after treatment (Fig. 4c). In infected mice, inflammatory cells started to infiltrate a large area of the lung, predominantly in the perivascular areas, at 2 to 7 dpi, expanding into the alveolar spaces with perivascular and interstitial edema at 7 dpi (Fig. 4c). SKAI-DS84 reduced the pulmonary inflammation at 2 and 7 dpi (Fig. 4c, Additional file 1: Fig. S4 Student’s t-test, *P* = 0.0337 and *P* = 0.0562, respectively). Notably, at 7 dpi, two out of five SKAI-DS84 treated mice showed complete resolution of pulmonary lesions. As reported previously, K18-hACE-2 mice infected with SARS-CoV-2 commonly developed splenic and intestinal lesions [18]. In the present study, two SARS-CoV-2-infected mice also showed moderate to severe white pulp atrophy with lymphoid apoptosis in the spleen at 7 dpi, whereas none of the SKAI-DS84-treated mice developed splenic lesions (Fig. 4d; one-way ANOVA, *P* = 0.0179). SKAI-DS84 also tended to reduce the duodenal atrophy observed at 7 dpi (Fig. 4e; one-way ANOVA, *P* = 0.02). Overall, the data from animal preclinical and in vitro experiments showed that SKAI-DS84 possesses the potential to inhibit infection by SARS-CoV-2 variants as a neutralizing antibody in vivo*.*

### SKAI-DS84 inhibits SARS-CoV-2 WT and variant infection by specifically binding to the quaternary epitopes of RBD and inhibiting the interaction of the RBD with hACE2

To directly assess the binding capability of antibodies to the RBD, we conducted protein thermal shift (PTS) and surface plasmon resonance (SPR) analyses. The PTS results illustrate the RBD-antibody binding efficiency up to a specific temperature, where higher dissociation temperatures indicate a stronger binding affinity between the antibody and the RBD. We investigated the melting temperature (Tm) values of SKAI-DS84 for both the B.1.617.2 and B.1.1.529 RBDs. When compared with the negative control group, SKAI-DS84 showed a Tm value of 56.4 °C in the PTS results for B.1.617.2, and a Tm value of 52.7 °C in the PTS results for the B.1.1.529 RBD. (Additional file 1: Fig. 3).

**Fig. 5:**
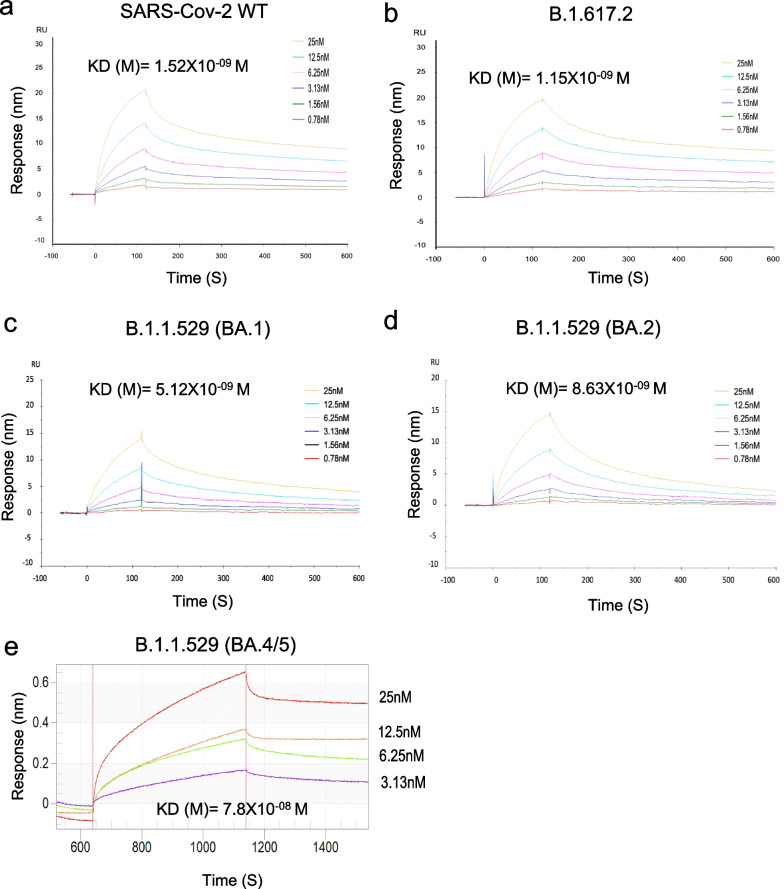
SKAI-DS84 may bind to the quaternary structure of the SARS-CoV-2 RBD. Surface plasmon resonance analysis of SKAI-DS84 against SARS-CoV-2 RBD and its variants. The RBDs of SARS-CoV-2 (**a**) WT, **b** B.1.617.2, **c** B.1.1.529 (BA.1), **d** BA.2, and **e** BA4/5 were immobilized on the biosensor. Subsequently, two-fold serially diluted SKAI-DS84 was allowed to flow over the biosensor surface. RBD, receptor-binding domain; WT, wild-type; SARS-CoV-2, severe acute respiratory syndrome coronavirus 2

We performed SRP analysis to evaluate the affinity of the SKAI-DS84 antibody for VOCs. As a result, high affinities for the RBD of WT (K_D_ = 1.52 nM), B.1.617.2 (K_D_ = 1.15 nM), B.1.1.529 BA.1 (K_D_ = 5.12 nM), and BA.2 (K_D_ = 8.63 nM) were observed. However, the BA.4/5 sub-variant of B.1.1.529 exhibited a 15-fold decrease in affinity compared with that of B.1.1.529 BA.1 (K_D_ = 78 nM) (Fig. 5).

**Fig. 6:**
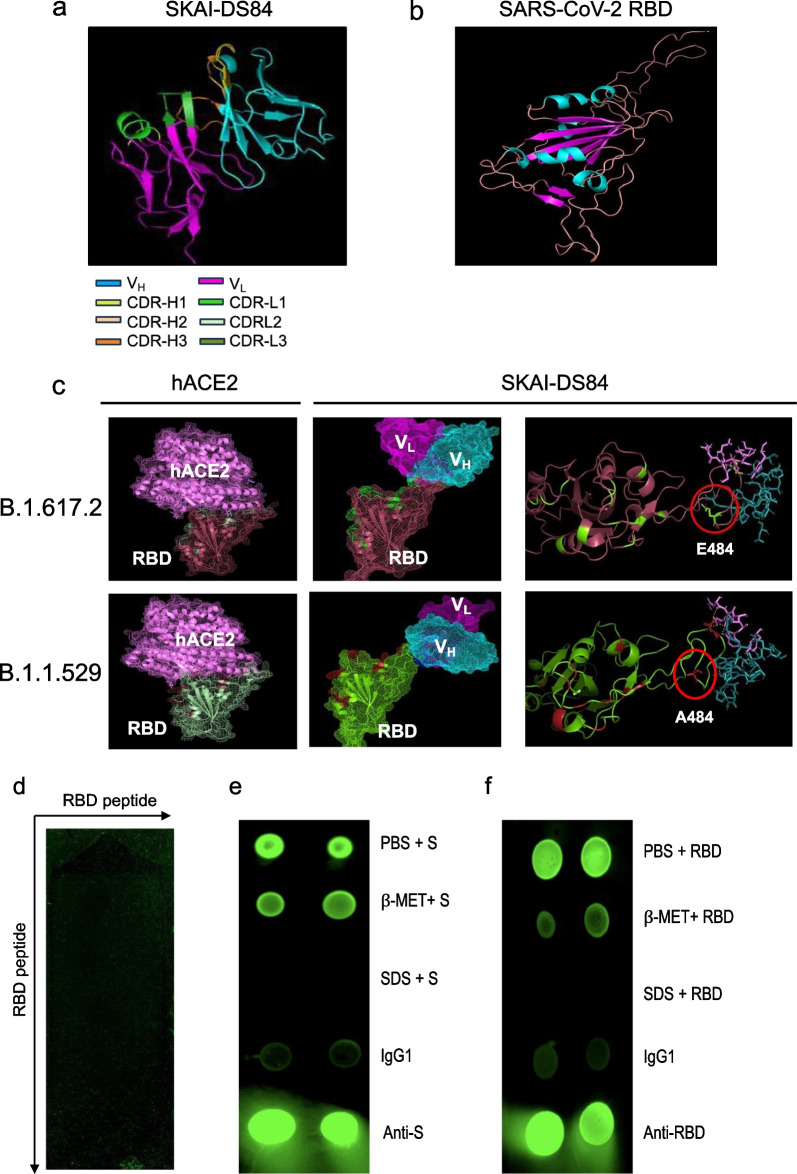
Identification of SKAI-DS84 binding mechanism. Structure of **a** SKAI-DS84 antibody and **b** RBD using in silico rosetta antibody **c** binding modeling of SKAI-DS84 antibody with B.1.617.2 and B.1.1.529 RBD using Cluspro2. **d** In total, 176,930 SARS-CoV-2 RBD residues consisting of 15 to 20 amino acid peptides were prepared on a chip and incubated with 1 ng/mL SKAI-DS84 to confirm antigen–antibody binding. The interaction (green) or lack of it (black) between RBD and SKAI-DS84 is shown by dots on the panels. **e**, **f** Quaternary epitope presentation by the SARS-CoV-2 RBD was assessed by testing for binding by representative quaternary epitope antibodies. Fluorescence-based dot blot analysis of quaternary SKAI-DS84 binding to either S or RBD proteins. SARS-CoV-2, severe acute respiratory syndrome coronavirus 2; RBD, receptor-binding domain

We elucidated the binding mechanism and structure of SKAI-DS84 and SARS-CoV-2 using in silico protein modeling techniques, namely Hex 8.0 ClusPro 2.0 server, sequence alignment, and antibody structure database. First, we performed homology modeling of the regions excluding HCDR3 using ClusPro. Subsequently, we re-modeled the HCDR3 region to predict the overall structure of SKAI-DS84 (Fig. 6a). Additionally, we utilized the cryo-EM structure [19] to model the structures of the SARS-CoV-2 B.1.617.2 and B.1.1.529 RBD, which will bind with SKAI-DS84 (Fig. 6b). We attempted to dock the SKAI-DS84 antibody onto the modeled proteins of the B.1.617.2 and B.1.1.529 RBD. As the four weak bonds involved in antigen–antibody binding (ionic interaction, hydrogen bond, hydrophobic interaction, Van der Waals force) occur within 5Å, we predicted the residues within the CDR region and within 5 Å, and proceeded with the binding modeling.

Based on the predicted binding modeling results, SKAI-DS84 directly binds to the SARS-CoV-2 RBD, blocking the binding with hACE2. Similar to the neutralization assay, SKAI-DS84 was found to more effectively inhibit binding with hACE2 in the B.1.617.2 compared to B1.1.529. This is due to the E484A substitution in the B.1.617.2, which reduces the polar interaction with R7 of CDR-H3, leading to a difference in binding angles. The binding angle between the B.1.617.2 RBD and SKAI-DS84 inhibits the efficient binding with hACE2 compared to the binding of the B1.1.529 RBD and SKAI-DS84. Furthermore, SKAI-DS84 CDR-H1 (Y3), HCDR2 (S7), and HCDR3 (Y10, D2, N10) regions are involved in polar interactions, and most LCDRs also participate in antigen–antibody binding. Moreover, SKAI-DS84 sufficiently exhibits neutralization efficacy against the B.1.1.529, indicating its ability to bind to RBD and block binding with hACE2. These interactions form a binding pocket that firmly grips the SARS-CoV-2 variants' RBD, effectively inhibiting their entry into host cells.

Through computer modeling, we indirectly demonstrated that SKAI-DS84 binds to the amino acid 484 of the RBD, specifically at CDR-H3 R7, which is associated with important sequences related to virus neutralization. Therefore, we conducted epitope mapping to directly confirm if SKAI-DS84 binds to specific regions of the SARS-CoV-2 RBD. To identify the epitope of the antigen that binds to SKAI-DS84, 176,930 peptides were individually synthesized, and 180,000 spots were identified on the microarray. The epitope mapping results confirmed that SKAI-DS84 did not bind to the peptides on the chip. Although the constructed peptides included all possible linear and conformational epitopes of the RBD sequence for each variant of the antigenic proteins, binding between SKAI-DS84 and the antigenic peptide was not confirmed (Fig. 6d). Thus, we hypothesized that SKAI-DS84 binds to a quaternary epitope formed by the interaction between RBDs and confirmed this using either S (Fig. 6e) or RBD (Fig. 6f) proteins. We used a recently published method to identify quaternary epitopes [20]. The binding between SKAI-DS84 and quaternary structure RBD was confirmed using PBS, β-mercaptoethanol, and sodium dodecyl sulfate (SDS). β-Mercaptoethanol breaks the disulfide bond, and SDS structurally denatures the protein. SKAI-DS84 exhibited complete antigen–antibody binding in the case of S or RBD treated with PBS. In the case of the antigen treated with β-mercaptoethanol, the binding force between the antibody and antigen was slightly reduced due to the disruption of the disulfide bonds. However, proteins completely denatured by SDS treatment did not show antigen–antibody binding. Ultimately, SKAI-DS84 engages in polar interactions within the RBD quaternary structure, forming a tight pocket structure that inhibits binding with hACE2, thereby suppressing cellular infection by SARS-CoV-2 WT and variants. Consequently, this mechanism effectively mitigates cellular infection by both the wild-type SARS-CoV-2 and its variants.

## Discussion

VOCs such as Alpha, B.1.617.2, and B.1.1.529 have emerged since 2019 and continue to evolve with new variants as of 2023 [21]. Therefore, this study aimed to develop neutralizing antibodies against both the SARS-CoV-2 WT and its variants. Previous studies have reported that antibody-based therapeutics can lose their neutralizing ability because of mutations in the target antigen [22]. In particular, mutations in the RBD of SARS-CoV-2, a single-stranded positive-sense RNA virus, naturally occur frequently [23]. These mutations, especially in the RBD, can make it challenging to develop neutralizing antibodies that target the RBD, as they can easily bypass the binding of antibodies, thereby rendering the role of antibody therapies ineffective, as widely known [24]. Thirty-one common mutations were found in the S protein of B.1.1.529, and S477R, Q498R, and N501Y substitutions increased the binding ability to the hACE2 receptor, while K417N increased the ability to avoid neutralizing antibodies [25]. Particularly, treatment with mAbs, such as bamlanivimab [26], etesevimab [27], casirivimab [28], and imdevimab [29], did not neutralize the variants. However, SKAI-DS84 exhibited slight variations in efficacy depending on the VOC, but it was able to neutralize not only the WT but also the currently circulating VOCs. We evaluated the potent neutralizing efficacy of SKAI-DS84 against the WT, B.1.617.2, and B.1.1.529 VOC variants using pseudovirus and authentic virus systems in vitro (Table 1). In contrast, bamlanivimab from Eli Lilly (Indianapolis, IN, USA) exhibited strong antiviral activity only against SARS-CoV-2 WT while showing no neutralizing efficacy against the B.1.1.529 variant and its subvariants. Additionally, we produced pseudoviruses for eight variants, including BA4/5, and tested SKAI-DS84 against them. SKAI-DS84 exhibited similar levels of neutralizing activity against seven of the variants but showed a 2–160-fold decrease in neutralization potency against BA4/5. According to recent reports, the hydrophobic interaction of F486V is reduced, resulting in a weaker binding affinity to SKAI-DS84, while the L452R and R493Q mutations increase the binding ability to hACE2 [30]. Therefore, the decrease in neutralization efficacy of SKAI-DS84 against BA4/5 is likely attributed to these changes.

Furthermore, according to in vivo results, when 50 mg/kg of SKAI-DS84 is injected into B.1.1.529-infected mice, it significantly inhibited viral activity, as confirmed by TCID_50_ and RT-qPCR. However, the lack of reduction in viral RNA levels on day 2 of infection was attributed to the characteristics of antibody therapy, where the antibodies bind to the viral surface and inhibit entry but are unable to suppress viruses already replicating intracellularly. On day 7, SKAI-DS84 circulating in the bloodstream prevented viral re-entry into cells, leading to viral clearance and decreased intracellular viral RNA levels (Fig. 4b). Additionally, it was observed that in B.1.1.529-infected mice treated with SKAI-DS84, pathogenicity in the lungs, duodenum, and spleen was restored to normal levels. However, the lack of reduction in viral RNA levels on day 2 of infection was attributed to the characteristics of antibody therapy, where the antibodies bind to the viral surface and inhibit entry but are unable to suppress viruses already replicating intracellularly [31]. On day 7, SKAI-DS84 circulating in the bloodstream prevented viral re-entry into cells, leading to viral clearance and decreased intracellular viral RNA levels (Fig. 4b). In the pharmacokinetics analysis, we collected blood samples and performed ELISA after intravenous administration of 10 or 50 mg/kg SKAI-DS84 in C57BL/6 mice. After intravenous injection of 10 and 50 mg/kg SKAI-DS84, T_max_ was reached after 24–96 h, suggesting absorption of SKAI-DS84 from the venous system into the systemic circulation (Additional file 1: Fig. S5). Additionally, it was observed that in B.1.1.529-infected mice treated with SKAI-DS84, pathogenicity in the lungs, duodenum, and spleen was restored to normal levels. In addition, using PTS, SPR, and ELISA analyses, we confirmed that SKAI-DS84 strongly binds to both variant and WT RBDs. In addition, using PTS, SPR, and ELISA analyses, we confirmed that SKAI-DS84 strongly binds to both variant and WT RBDs. Furthermore, we confirmed that SKAI-DS84 does not bind to the RBD in the liner structure of the protein. We demonstrated that SKAI-DS84 binds to the quaternary structure of the epitope [21] using a protein-denaturing assay, making it of particular interest by revealing a novel SARS-CoV-2 inhibitory mechanism. This considerably enhances the binding affinity of the full antibody as it enables binding to all three RBD subunits in the S protein trimer. Therefore, we speculated that SKAI-DS84 shows a strong neutralizing effect on VOCs as well as WT SARS-CoV-2. Despite the ability of SKAI-DS84 to neutralize SARS-CoV-2 VOCs, understanding the basic mechanism that interferes with the binding between the quaternary structure of RBD and hACE2 is lacking. However, this antibody may facilitate the elucidation of the molecular interaction between the unknown RBD quaternary structure and hACE2.

ADE analysis [32, 33] using pseudoviruses did not provide any evidence regarding SKAI-DS84-dependent viral infection in FcRI- and II-expressing cells (Raji and U937 cells), consistent with the lack of exacerbation of symptoms in experiments with in vivo data. Moreover, a recent animal study reported that ADE was not observed in a vaccine-targeted SARS-CoV-2 RBD group. These observations suggested that SKAI-DS84 can bind to the RBD and interfere with the formation of quaternary structures, thereby neutralizing SARS-CoV-2 and alleviating pathological symptoms without ADE in mouse preclinical studies [12, 15].

The main limitation of our study is the lack of activity of SKAI-DS84 against subvariants such as BL3 and ABL3 (including XBB1.5), as well as the lack of preclinical animal efficacy data due to inadequate infrastructure. However, we attempted to overcome these limitations by utilizing pseudoviruses to validate the neutralizing capacity of SKAI-DS84. Furthermore, by elucidating the binding mechanism to the quaternary structure of the epitope, SKAI-DS84 may demonstrate therapeutic value against emerging B.1.1.529 subvariants. Additionally, combination therapy with SKAI-DS84 and FDA-approved antiviral drugs, such as remdesivir, molnupiravir, and nirmatrelvir, can provide a potent antiviral strategy for eliminating emerging B.1.1.529 subvariants.

## Conclusion

We identified the SKAI-DS84 antibody through phage display technology, and SKAI-DS84 has demonstrated neutralizing effects not only against SARS-CoV-2 WT but also broad SARS-Cov-2 variants. The neutralization mechanism of SKAI-DS84 may be associated with the close pocket configuration formed by antigen–antibody polar interactions within the complete quaternary structure of the RBD. This suggests that SKAI-DS84 potentially inhibits SARS-CoV-2 infection by binding to the RBD of SARS-CoV-2, thus interfering with its binding to the hACE2 receptor.

### Supplementary Information

**Additional file 1.** Additional material and figures.

## Data Availability

Not applicable.

## Abbreviations

ADE: Antibody-dependent enhancement
BSL 3: Biosafety level 3
CDR: Complementarity-determining region
DPI: Day post infection
EDC: 1-Ethyl-3-(3-dimethylaminopropyl)-carbodiimide
ELISA: Enzyme-linked immunosorbent assay
EUA: Emergency use authorization
FBS: Fetal bovine serum
GAPDH: Glyceraldehyde-3-phosphate dehydrogenase
hACE2: Human angiotensin-converting enzyme 2
IgG1: Immunoglobulin G1
K_D_: Disassociation constant
KCDC: Korea Centers for Disease Control and Prevention
mAb: Monoclonal antibody
NHS: N-hydroxysuccinimide
PEG: Polyethylene glycerol
RBD: Receptor-binding domain
scFv: Single-chain fragment variable
SARS-COV-2: Severe acute respiratory syndrome coronavirus 2
S protein: Spike protein
SPR: Surface plasmon response
VOI: Variant of infection
VOC: Variant of concern
WHO: World Health Organization
WT: Wild-type
WST: Water soluble tetrazolium
