# Supplementary material for: Development of a neutralization monoclonal antibody with a broad neutralizing effect against SARS-CoV-2 variants
Source: Virol J. 2023 Dec 1;20:285. doi: 10.1186/s12985-023-02230-9 (PMC10693169; doi:10.1186/s12985-023-02230-9)
Supplement: Supplementary file 1 — Additional file 1. Additional material and figures. [file 12985_2023_2230_MOESM1_ESM.docx]

**Additional Files**

**Development of a SARS-CoV-2 cross-neutralization antibody with a potent neutralizing effect against SARS-CoV-2 variants**

Hae Li Ko^a¶^, Deuk-ki Lee^a¶^, Younghyeon Kim^a,b^, Hui Jeong Jang^c^, Youn Woo Lee^c^, Sang-Hyuk Seok^d^, Jin-Kyung Limb^e^, Da In On^e,f^, Jun-Won Yun^f^, Jun Won Park^d^, Ho-Young Lee^c^, Je Kyung Seong^e,f,h^*and Sungjin Lee^a^*

**Additional materials**

| **REAGENT or RESOURCE** | **SOURCE** | **IDENTIFIER** |  |
| --- | --- | --- | --- |
| **Plasmid** |  |  |  |
| OPAL library | Ewha Woman University | N/A |  |
| pMD2.G | addgene | Cat#12259 |  |
| Plasmid encoding the SARS-CoV-2 S protein | The Rockefeller University | N/A |  |
| pLenti-SFFV-NanoLuc_PGK_RPF_T2A_PURO Lentiviral Reporter Plasmid | ALSTEM | Cat#LR442 |  |
| Spike pseudotyping plasmid – Indian (delta) Variant | InvivoGen | Cat#pLV-SpikeV8 |  |
| Spike pseudotyping plasmid – Omicron Variant (B.1.1.529/BA.1) | InvivoGen | Cat#pLV-SpikeV11 |  |
| psPAX2 | addgene | Cat#12260 |  |
| TGEX_HC Expression Vector | Antibody Design | Cat#MX001 |  |
| TGEX_LC Expression Vector | | Antibody Design | Cat#MX002 |
|  |  |  |  |
| **Medium and supplement** |  |  |  |
| ExpiCHO^TM^ Expression Medium | Thermo Fisher Scientific | Cat#A1435102 |  |
| Fetal bovine serum | Gibco | Cat#16000 |  |
| L-glutamine | Cytiva | Cat#SH30852.01 |  |
| Non-essential-amino acid | Cytiva | SH30853.01 |  |
| Penicillin-streptomycin | Cytiva | Cat#SV30010 |  |
| Puromycin Dihydrochloride | Gibco | Cat#A1113803 |  |
| RPMI-1640 | Cytiva | SH30096.FS |  |
|  |  |  |  |
| **Protein and Antibody** |  |  |  |
| Bamlanivimab Biosimilar-Anti-Covid spike RBD mAb | ProteoGenix | Cat#PX-TA1031-100ug |  |
| Recombinant SARS-CoV-2 B.1.617.2 Spike RBD His Protein | R&D systems | Cat#10876-CV |  |
| Recombinant SARS-CoV-2 B.1.1.529 S RBD His-taq Protein | | R&D systems | Cat#11056-CV |
| RecombinantSARS-CoV-2 Spike RBD His protein | R&D systems | Cat#10500-CV |  |
| SARS-CoV-2 Spike RBD Protein, His Taq (BA.4&BA.5/Omicron)(MALS verified) | Acro biosystems | Cat#SPN-C522e-50ug |  |
| SARS-CoV-2 Spike RBD, His Taq (BA.2/Omicron)(MALS verified) | Acro biosystems | Cat#SPD-C5222-100ug |  |
| SARS-CoV-2 B.1.1.529 (Omicron) Spike RBD Protein (His taq) | SinoBiological | Cat#40592-V08H121 |  |
|  |  |  |  |
| **Reagents and Materials** |  |  |  |
| 2x Yeast Extract Tryptone | BD | Cat#11738892 |  |
| Bsal-HF®v2 | NEB | Cat#R3733L |  |
| Bsmbl-v2 | NEB | Cat#R0739L |  |
| BspEl | NEB | Cat#R0540L |  |
| BssHll | NEB | Cat#R0199L |  |
| Dynabeads^TM^ M-270 Epoxy | Invitrogen | Cat#14301 |  |
| Econo-Pac® Chromatography Columns | Bio-rad | Cat#7321010 |  |
| High 10^8^ HIT-DH5α | RBC | Cat#RH618 |  |
| Lenti-X^TM^ Concentrator | TAKARA | Ct#631232 |  |
| Lipofectamine^TM^ 3000 Transfection Reagent | Invitrogen | Cat#L3000015 |  |
| High 10^8^ HIT-DH5α | RBC | Cat#RH618 |  |
| Polyethylene glycerol 8000 | Sigma-Aldrich | Cat#P2139 |  |
| rProtein A Agarose Resin | Amicogen | Cat#1011000 |  |
| Sensor Chip CM5 | Cytiva | Cat#29149603 |  |
|  |  |  |  |
| **Assay kit** |  |  |  |
| Amine Coupling Kit | Cytiva | Cat#BR100050 |  |
| EZ_Cytox | Gogenbio | Cat#EZ-5000 |  |
| MagMAX^TM^ *mir*Vana^TM^ Total RNA Isolation Kit | Applied Biosystems | Cat#A27828 |  |
| Nano-Glo® Luciferase Assay System | Promega | Cat#N1130 |  |
| Protein Thermal Shift^TM^ Starter kit | Applied Biosystems | Cat#4462263 |  |
| RNeasy® mini kit | Qiagen | Cat#74104 |  |
| SARS-CoV-2 Surrogate Virus Neutralization Test (sVNT) Kit | Genscript | Cat#L00847 |  |
| QuantiFast STBR Green RT-PCR kit | Qiagen | Cat#204156 |  |

**Additional Figure 1**

**Identification of antibodies for targeting the RBD S protein of SARS-CoV-2 VOC by bio-panning.** (a) Schematic of the bio-panning process. (b) Verification of the ability of the polyclonal scFv phage to bind to the B.1.617.2 RBD of SARS-CoV-2 using phage groups after five rounds. (c) Confirmation of binding ability through ELISA of monoclonal scFv phage of the 5th bio-panning. SARS-CoV-2, severe acute respiratory syndrome coronavirus 2; RBD, receptor-binding domain; S, spike; VOC, variant of concern.

**Additional Figure 2**


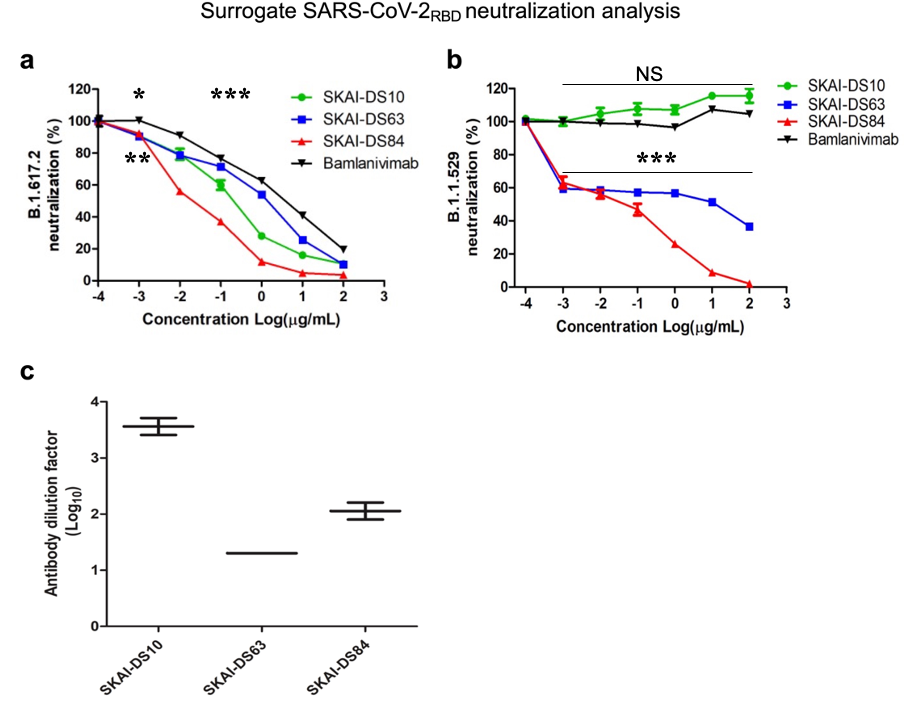


**Neutralization effect of SKAI-DS10, SKAI-DS63, and SKAI-DS84 on surrogate neutralization kits and authentic viruses.** Dose-dependent effects of SKAI-DS10, SKAI-DS63, SKAI-DS84, and bamlanivimab on SARS-CoV-2. (a) B.1.612.7 and (b) B.1.1.529 neutralization were determined by measuring the relative inhibition of SARS-CoV-2 RBD–hACE2 interaction with increasing concentrations of either SKAI-DS10, SKAI-DS63, SKAI-DS84, or bamlanivimab. (c) The data show the neutralizing effect of SKAI-DS10, SKAI-DS63, and SKAI-DS84 on the SARS-CoV-2 authentic virus in VeroE6 cells. SARS-CoV-2, severe acute respiratory syndrome coronavirus 2; RBD, receptor-binding domain. * P < 0.05, ** P < 0.01, significantly different from control.

**Additional Figure 3**


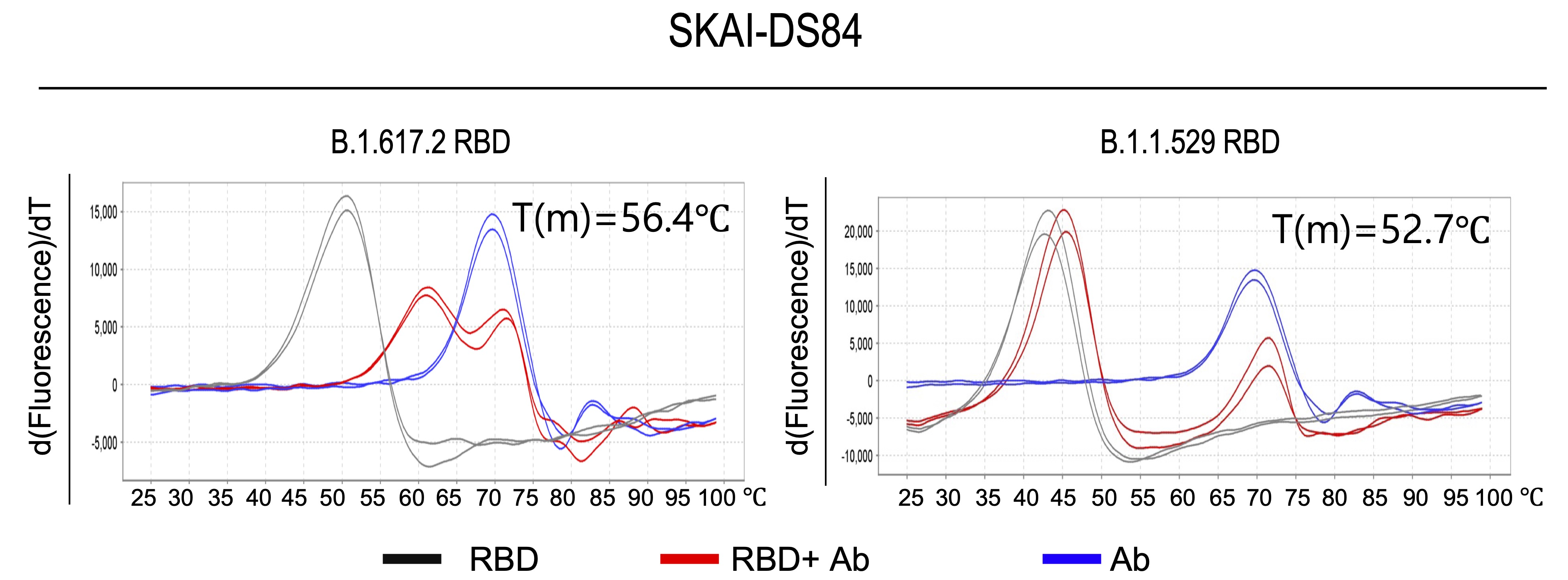


**The fluorescence-based thermal shift assay for protein binding to SKAI-DS10, SKAI-DS63, and SKAI-DS84.** The curve shows a shift as the T_m_ increases when the protein binds to the antibody. Black; SARS-CoV-2 VOC RBD protein only, Red; SARS-CoV-2 VOC RBD protein with SKAI-DS84, Blue; SKAI-DS84 only. SARS-CoV-2, severe acute respiratory syndrome coronavirus 2, RBD, receptor-binding domain; T_m_, melting temperature; VOC, variant of concern.

**Additional Figure 4**

**Results of the scores for inflammation and edema in the lungs after SKAI-DS84 treatment in B.1.1.529-infected K18-hACE2 expressing**

|  |  | Dpi | Inflammation | Edema |
| --- | --- | --- | --- | --- |
| No infection | SB41,42-N1 | 7 | 0.5 | 0 |
|  | SB41,42-N2 | 7 | 0 | 0 |
|  | SB41,42-N3 | 7 | 0 | 0 |
|  | SB41,42-N4 | 7 | 0 | 0 |
| W/O Ab | SB41,42-V1 | 2 | 1.5 | 2.5 |
|  | SB41,42-V2 | 2 | 2 | 2 |
|  | SB41,42-V3 | 2 | 2 | 2 |
|  | SB41,42-V4 | 2 | 2 | 1.5 |
|  | SB41,42-V5 | 2 | 1.5 | 1 |
|  | SB41,42-V6 | 7 | 4.5 | 3 |
|  | SB41,42-V7 | 7 | 4 | 3 |
|  | SB41,42-V8 | 7 | 4.5 | 2.5 |
|  | SB41,42-V9 | 7 | 3 | 2.5 |
|  | SB41,42-V10 | 7 | 3 | 2.5 |
| SKAI-DS84 | SB41-TH1 | 2 | 1.5 | 1.5 |
|  | SB41-TH2 | 2 | 1 | 1.5 |
|  | SB41-TH3 | 2 | 1.5 | 1.5 |
|  | SB41-TH4 | 2 | 0.5 | 0.5 |
|  | SB41-TH5 | 2 | 1.5 | 1.5 |
|  | SB41-TH6 | 7 | 3.5 | 2.5 |
|  | SB41-TH7 | 7 | 3 | 3 |
|  | SB41-TH8 | 7 | 0 | 0.5 |
|  | SB41-TH9 | 7 | 0 | 0 |
|  | SB41-TH10 | 7 | 3 | 2.5 |

**Additional Figure 5**


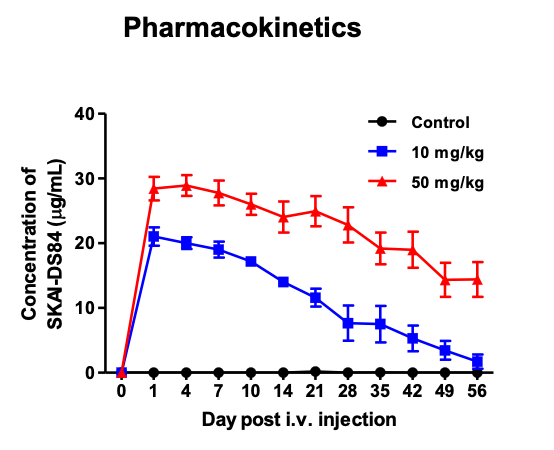


**Pharmacokinetic analysis of the SKAI-DS84 antibody.** Pharmacokinetic analysis of human IgG Ab levels over time after intravenous administration of 10 and 50 mg/kg SKAI-DS84. SARS-CoV-2
